# The Development of a Coaxial Electrospinning Formula Using Fish Gelatin/PBS as the Core for Structurally Intact Liposome Loading and Release

**DOI:** 10.3390/polym17070944

**Published:** 2025-03-31

**Authors:** Haoyu Wang, Runnan Xia, Mo Zhou, Gareth R. Williams, Evzen Amler, Feng-Lei Zhou, Maryam Tamaddon, Chaozong Liu

**Affiliations:** 1 Division of Surgery & Interventional Science, University College London, Royal National Orthopaedic Hospital, Stanmore HA7 4LP, UK; haoyu.wang@ucl.ac.uk (H.W.); runnan.xia.22@alumni.ucl.ac.uk (R.X.); mo.zhou.20@ucl.ac.uk (M.Z.); m.tamaddon@ucl.ac.uk (M.T.); 2 UCL School of Pharmacy, University College London, 29-39 Brunswick Square, London WC1N 1AX, UK; g.williams@ucl.ac.uk; 3 University Centre for Energy Efficient Buildings, Czech Technical University in Prague, Trinecka 1024, 273 43 Bustehrad, Czech Republic; amler@seznam.cz; 4 Centre for Medical Image Computing, Department of Medical Physics and Biomedical Engineering, University College London, London WC1V 6LJ, UK; fenglei.zhou@ucl.ac.uk; 5 College of Textiles and Clothing, Qingdao University, Qingdao 266071, China

**Keywords:** coaxial electrospinning, fish gelatin, PCL, liposomes

## Abstract

In electrospun scaffolds, coaxial electrospinning is gaining increased attention due to its potential for biocomponent encapsulation and controlled delivery. However, the encapsulation of biocomponents, such as liposomes, remains challenging because of their low stability in commonly used electrospinning solvents. This study, therefore, aims to develop a novel coaxial electrospinning formulation for crafting a liposome-encapsulated, rapid-release coaxial fiber. Liposomes demonstrated desirable stability in fish gelatin/phosphate-buffered saline (PBS) solutions, which remain liquid at room temperature and exhibit exceptional spinnability at concentrations exceeding 80 *w*/*v*% due to the reduction in surface tension. Fluorescent labelling examinations confirmed the successful encapsulation of liposomes within coaxial fibers electrospun from a 160 *w*/*v*% gelatin/PBS core and a 20 *w*/*v*% PCL/chloroform/N,N-dimethylformamide (DMF) shell. The gelatin/PBS core solution formed solid ends at the tips of the core-shell fiber post-spinning, while maintaining a liquid state within the shell, thereby enabling the encapsulation of liposomes within the PCL coaxial fiber. Upon exposure to medium, the solid ends dissolve, enabling the rapid release of liposomes. The successful development of this liposome-loaded electrospun coaxial fiber, using fish gelatin, highlights its potential for creating advanced liposome delivery systems.

## 1. Introduction

Electrospinning is a technique used to generate nano- to micro-scale fibers [1], and it is therefore applied in the fabrication of extracellular matrix (ECM)-mimicking scaffolds [2,3]. These scaffolds have the ability to respond to the local environment [4,5] and provide cues for cell differentiation after implantation [6,7], achieved by delivering bioactive components such as anti-inflammatory drugs or cytokine factors through the loading of bioactive components [8,9] or with their carriers, such as liposomes [10,11].

Liposomes, as commonly used drug carriers, offer several advantages, including compatibility with both hydrophilic and lipophilic drugs, prolonged release profiles, and desirable biocompatibility and biodegradability [12]. However, concerns have been raised during the development of liposome-loaded electrospun fibers, particularly regarding stability during direct blending due to exposure to organic solvents and potential configuration shifts.

Methods to stabilize the integration between liposomes and electrospun fibers have been proposed, such as physical adsorption [13,14], chemical conjugation [15], and biological affinity [16]. These approaches aim to maintain liposomal structural integrity via exposure to storage media. This also raises concerns about drug loss during long-term storage.

Coaxial electrospinning is a promising method for liposome loading, as the shell can act as a barrier. In this method, liposomes are loaded into the core, thus avoiding exposure to external environments such as storage media. Evzen’s group (2012) [17] was the first to propose coaxial electrospinning for liposome encapsulation as a drug-loading system. They used polyvinyl alcohol (PVA) as the core and poly-ε-caprolactone (PCL) as the shell. Despite advancements over the past decade [17,18,19,20,21,22], formulations for encapsulating liposomes via coaxial electrospinning remain limited.

However, most existing coaxial electrospinning formulations have limitations in maintaining liposome structural integrity. For example, the half-life for soybean-derived l-α-phosphatidylcholine-based liposomes in PVA/Tris-buffered saline is within 24 h, depending on the concentration of core solutions [17]. This highlights a critical gap in the selection of core materials that provide both electrospinnability and liposome stability. Thus, expanding the limited range of viable core solutions [17,18,19,20,21,22] remains a challenge in coaxial electrospinning for drug delivery applications.

Gelatin, a hydrolysate of collagen [23], has been widely investigated for liposome stabilization in hydrogel matrices [24,25,26,27]. However, its use in electrospinning has been hindered by its strong gelation tendency, which reduces solution fluidity and limits its electrospinnability [28]. This challenge is particularly pronounced in mammalian-derived gelatin, which undergoes α-helix formation via hydrogen bonding at temperatures below 40 °C, causing gelation and poor processability [23,28,29,30]. To counteract this, past studies have relied on strong organic solvents such as hexafluoroisopropanol [31], 2,2,2-trifluoroethanol [32,33], or acids [32,34,35] to dissolve gelatin and maintain electrospinnability. However, these solvents can severely compromise liposome stability, leading to membrane disruption or aggregation.

In this study, a fish gelatin aqueous solution, rather than the commonly used organic solvents, was novelly used as the core material for coaxial electrospinning. Unlike mammalian gelatin, fish gelatin exhibits reduced gelation tendencies at room temperature due to its lower proline and hydroxyproline content [36,37,38]. However, using fish gelatin as an aqueous solution for electrospinning has never been reported, as its high surface tension counteracts cohesive forces, leading to the breakdown of the coaxial structure at commonly used concentrations (<40 *w*/*v*%).

We first report that fish gelatin remains fluid even at high concentrations (>200 *w*/*v*%) without requiring organic solvents, thereby eliminating solvent-induced liposome damage. Furthermore, a significant reduction in surface tension was observed at fish gelatin/PBS concentrations exceeding 80 *w*/*v*%, which dramatically improved spinnability. By leveraging this enhanced processability and liposome compatibility, we successfully developed a coaxial electrospinning formulation using a fish gelatin/PBS core and a PCL/chloroform/DMF shell for stable liposome encapsulation. Our approach preserves the structural integrity of liposomes throughout the electrospinning process and enables their rapid release upon exposure to aqueous environments.

## 2. Materials and Methodology

### 2.1. Materials

Polycaprolactone (PCL, Mw = 80,000), fish gelatin (Mol wt = 60 kDa), and L-α-phosphatidylcholine were purchased from Sigma Aldrich (Dorset, UK) and used as received. The solvents N, N-dimethylformamide (DMF), chloroform, phosphate-buffered saline (PBS, pH 7.2), and the fluorescent dye fluorescein isothiocyanate (FITC) were also procured from Sigma-Aldrich. Cell culture consumables included a live/dead kit (calcein-AM/ethidium homodimer), penicillin/streptomycin (P/S) solution, fetal bovine serum (FBS), and Prestoblue^tm^ and were purchased from Thermofisher (Loughborough, UK). Endothelial cell growth medium was purchased from PromoCell (Heidelberg, Germany).

### 2.2. Solution Preparation

PCL was dissolved in a mixture of chloroform and DMF (4/1, *v*/*v*) with concentrations ranging from 1 *w*/*v*% to 25 *w*/*v*%, with increments of 2 *w*/*v*%. The mixture was then stirred continuously using a magnetic stirrer set to 100 rpm. This process was conducted at room temperature and continued overnight to ensure complete dissolution of the PCL. Gelatin derived from cold water fish was prepared by dissolving it in 1× phosphate buffered saline (PBS) with concentrations ranging from 20 *w*/*v*% to 200 *w*/*v*%, with increments of 20 *w*/*v*%. This process was carried out using a heating block set to maintain a constant temperature of 60 °C overnight to achieve homogeneity. The solution was used within one week and stored in a fridge at 4 °C. Surface tension was measured using a bubble pressure tensiometer (Krüss, Germany) at 100 ms intervals (*n* = 3).

### 2.3. Set-Up

The electrospinning system consisted of a syringe pump (Model 300, WPI, Hertfordshire, UK), a single needle (stainless steel, 19G) or a coaxial needle (stainless steel, 21G/19G), a high voltage generator (40 KV, Linari, Italy), and a stainless steel drum (D = 1), which was mounted to a motor set to 100 rpm to collect the electrospun coaxial fiber. The electrospinning voltage remained at 18 kV, and the distance remained at 15 cm. The fabrication was performed in a room with ambient conditions.

#### 2.3.1. Coaxial Formula Optimization

The effects of the PCL and gelatin concentrations and the feed rate (gelatin/PCL, *v*/*v*) on the formation of coaxial fibers were evaluated as described in Table 1. Specifically, concentrations of 15, 20, and 25 *w*/*v*% for PCL in chloroform/DMF (4/1, *v*/*v*), 120, 160, and 200 *w*/*v*% for gelatin/PBS, and feed ratios of 1:1, 1:2.5, and 1:5 were chosen to investigate the effect of these three factors on the spinnability.

#### 2.3.2. Coaxial Fiber Encapsulate and Release Liposomes and FITC

The combination of 160 *w*/*v*% gelatin, 20 *w*/*v*% PCL, and a feed ratio of 1/2.5 *v*/*v* was selected for the liposome-loaded gelatin/PCL core/shell fiber fabrication, due to good spinnability and less sub-Taylor cone formation. FITC, used as the drug model, was loaded into both the core and shell to prepare FITC + gelatin/PCL and gelatin/PCL + FITC fibers, respectively. The release processes of FITC and liposomes from these coaxial fibers were then studied.

Liposome preparation was prepared via the hydration method [17]. In detail, 25 mg of soybean-derived L-α-phosphatidylcholine was dissolved in 1 mL of chloroform in a flask mounted on a rotary evaporator, mixed with 0.1 mM Nile Red for visualization, to form a thin lipid film. Then, 10 mL of PBS was added to the dried flask and hydrated in an incubator for 4 h at 100 rpm. A liposome suspension was obtained and stored in the refrigerator before use.

The diameter of the liposomes was determined using optical microscopy (Zeiss, Axioscope 5, Jena, Germany). Liposome suspensions were imaged in bright-field mode, and the size distribution was analyzed using ImageJ software (Version 6.0, NIH, Bethesda, MD, USA). A total of 100 liposomes from the acquired images were measured to calculate the average diameter and standard deviation. (1.2) To prepare Nile Red-labeled liposome + gelatin/PCL core-shell fiber, 5 mL of a 160 *w*/*v*% liposome-gelatin mixture was used as the core solution for coaxial electrospinning. The core solution was made from a mixture of 4 mL of a 200 *w*/*v*% gelatin solution mixed with 1 mL of a liposome suspension. The release of these liposomes was observed under a fluorescent microscope (Zeiss, Axioscope 5, Germany). The as-spun coaxial fiber was collected on slides for a quick check. Then, the fibers were cryo-breaked and immersed in 10 mL PBS in a Petri dish and continuously imaged for 2 min at 12 frames/min to visualize the release of the liposomes.

The release of the model drug fluorescein (FITC) from the gelatin core and shell was assessed using a Tecan Infinite^®^ M200 PRO microplate reader (Tecan Group Ltd., Männedorf, Switzerland) in fluorescence mode. Coaxial fiber loaded with FITC was cut into round patches (10 mm diameter) and incubated with 50 mL of PBS at room temperature. 150 μL of PBS was withdrawn periodically, and fluorescence intensity was measured at an excitation wavelength of 490 nm and emission wavelength of 540 nm (*n* = 5). A standard curve was prepared by serial dilution of a known concentration of FITC in the same medium used for the standard curve plotting, with a concentration range of 0.1 µg/mL to 10 µg/mL.

#### 2.3.3. Coaxial Fiber Characterization

The morphology and topography of the samples were observed using scanning electron microscopy (SEM, Phenom ProX, Thermo Fisher Scientific, Waltham, MA, USA) with an accelerating voltage of 5 kV. To examine the core–shell structure, the fibers were frozen and fractured in liquid nitrogen. All samples were dried in a fume hood then subjected to gold sputtering, SEM imaging, and analyzed using ImageJ software version 1.8.0 (NIH, USA) to assess morphological features such as fiber diameter, wall size, and core area at 100, 50, and 50 randomly selected points on captured SEM images, respectively.

Samples were cut into 0.5 × 0.5 cm squares and mounted on a glass slide. Changes in the water contact angle were captured via optical lenses (Ossila, Sheffield, UK) and compared with neat 20 *w*/*v*% electrospun PCL fiber. A 10 µL droplet of distilled water was placed on the surface, and its profile was captured for 1 min at 120 frames/min. The images were analyzed using the manufacturer-provided software. This process was repeated 5 times.

Tensile tests were performed with a Zwick 0.5 universal tensile test instrument (Zwick Roell, Ulm, Germany) to evaluate the mechanical properties of the fibrous matrix. The samples were cut into 3 cm × 0.4 cm sheets with 100 μm thickness. The test followed DIN EN ISO 527-1, and Young’s modulus, maximum strength, and strain at break were recorded with the Zwick/Roll software (testXpert, II V3.0, Zwick Roell, Ulm, Germany) automatically (*n* = 5).

For Fourier-transform infrared spectroscopy (FTIR, Perkin Elmer 2000, Springfield, IL, USA), samples were prepared by placing 0.5 × 0.5 cm pieces onto the sample platform. The spectra were recorded over a wavelength range of 4000 to 450 cm^−1^ with a resolution of 1 cm^−1^. Baseline correction and normalization procedures were applied to each spectrum for consistency in peak analysis.

#### 2.3.4. In Vitro Evaluation

Human Umbilical Vein Endothelial cells (HUVECs, PromoCell, Heidelberg, Germany) were selected and cultured following the supplier’s protocol. In brief, cells were cultured with endothelial cell growth basal medium under standard conditions. Passage 5-10 HUVECs were detached using 0.25% trypsin-EDTA solution, then centrifuged and resuspended in fresh medium to achieve a seeding density of 30,000 cells per well. A 10 μL cell suspension droplet was seeded over the scaffold’s inner surface. After 4 h of cell attachment, 400 μL of fresh medium was added to the well.

Cell viability was tested via PrestoBlue on days (D) 1, 4, and 7 as well as the Live/Dead assay. The Live/Dead kit was prepared as a staining solution with 5 mM calcein-AM and 2 mM ethidium homodimer dissolved in PBS solution to visualize live cells in green and dead cells in red. The cell-seeded fibers on D4 and D7 were soaked in the staining solution at room temperature for 15 min in the dark and observed under a fluorescent microscope (Zeiss, Axioscope 5, Germany).

#### 2.3.5. Statistical Analysis

The results are presented as mean ± standard deviation. Statistical analyses and plotting were conducted using Origin software (Origin Pro 2024B, OriginLab, Northampton, MA, USA). For comparisons involving the fiber diameter, wall thickness, and pore size, either a T-test or a Mann–Whitney test was applied, depending on the results of the D’Agostino–Pearson normality test. For cell proliferation experiments, comparisons were made between all values and the control (D1) with cells seeded on a tissue culture plate (TCP). Two-way analysis of variance (ANOVA) was used to analyze the significance level of differences between timepoints and groups, and post-hoc comparisons were performed using the Tukey’s Honest Significant Difference (HSD) test, with results displayed as *p*-values, where *, **, and *** correspond to *p* < 0.05, *p* < 0.01, and *p* < 0.001, respectively.

## 3. Results

### 3.1. Coaxial Formula Development

**Figure 1:**
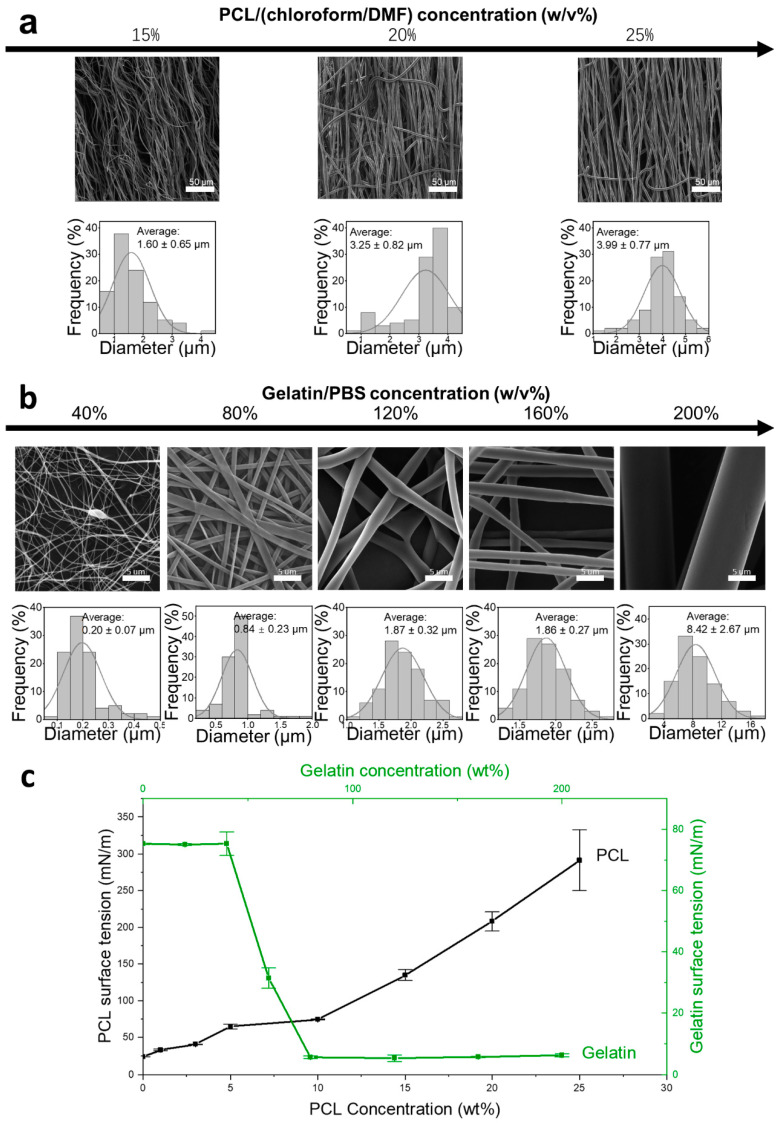
Exploration of the aqueous gelatin core-based coaxial electrospinning formula. Variation in spinnability of (**a**) PCL/chloroform/DMF with concentrations ranging from 15 to 25 *w*/*v*%, and (**b**) gelatin/PBS, with concentrations from 40 to 200 *w*/*v*% analyzed through SEM images to determine the corresponding fiber diameters (*n* = 100). (**c**) A dual-axis line graph illustrating the relationship between surface tension and concentration for both PCL and Gelatin solutions. The left Y-axis and bottom X-axis detail the surface tension of PCL, starting from 0 *w*/*v*% (pure chloroform/DMF mixture), while the right Y-axis and top X-axis display the gelatin solution’s surface tension starting from 0 *w*/*v*% (pure PBS solution). Each configuration was tested thrice (*n* = 3).

PCL/chloroform/DMF shell solutions and gelatin/PBS core solutions were first independently processed to assess their spinnability; the variation in fiber formation with concentration is shown in Figure 1. It is revealed that both PCL and gelatin exhibited a wide range of spinnability, with PCL concentrations ranging from 15 *w*/*v*% to 25 *w*/*v*% (Figure 1a), and gelatin from 40 *w*/*v*% to 200 *w*/*v*% (Figure 1b), that covering the intended concentrations for coaxial formulation. Fish gelatin dissolved in PBS exhibited exceptional spinnability at room temperature. The fiber diameters varied from nanoscale (at 40 *w*/*v*%, averaging 0.20 ± 0.07 μm) to microscale (at 200 *w*/*v*%, averaging 8.42 ± 2.67 μm), and demonstrated different morphologies: bead structures at concentrations of 40 *w*/*v*%, smooth fibers between 40 *w*/*v*% and 160 *w*/*v*%, and buckled fibers at concentration of 200 *w*/*v*%. This remarkable spinnability can be attributed to the significant reduction in surface tension at higher concentrations (Figure 1c). Initially, the surface tension of gelatin in PBS remained approximately stable at lower concentrations, around the value of PBS itself, 75.23 ± 0.25 mN/m, then significantly decreased to 5.53 ± 0.42 mN/m at 80 *w*/*v*% concentration and thereafter stabilized. By contrast, the surface tension of PCL in a chloroform/DMF mixture solvent significantly increased from 23.76 ± 0.05 mN/m to 291.2 ± 41.17 mN/m when the concentration increased from 1 *w*/*v*% to 25 *w*/*v*%. The higher surface tension of PCL, acting as the shell component, suggests that PCL will readily envelop and encapsulate the gelatin core.

**Figure 2:**
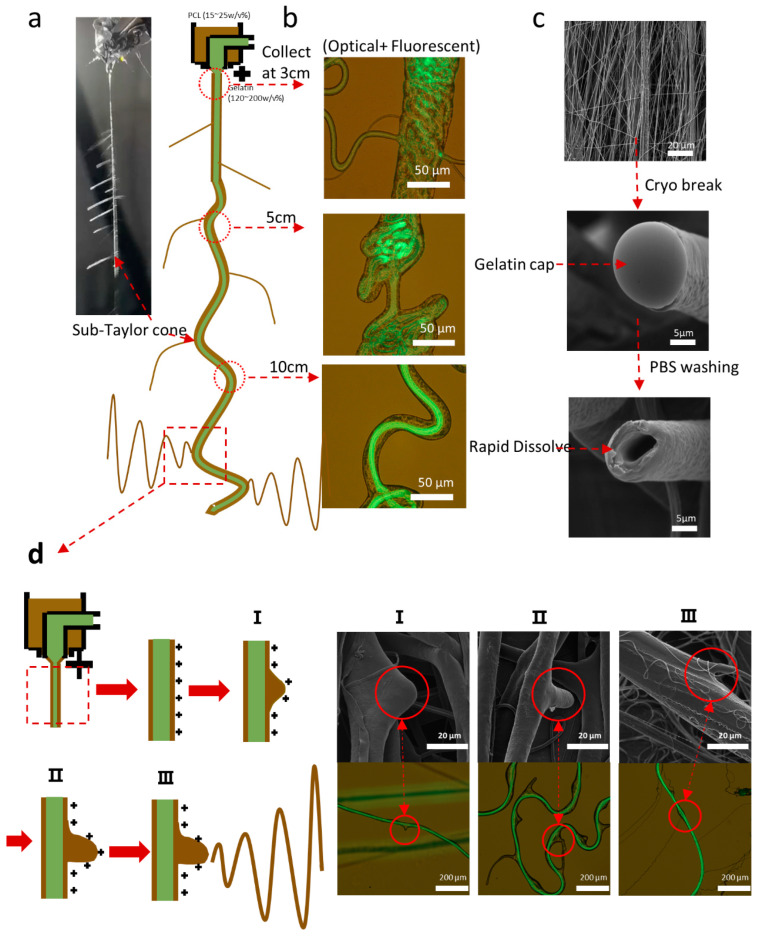
Recapitulation of characteristics in the coaxial electrospinning process and fiber formation. (**a**) A real-time photograph alongside an illustrative sketch of the fiber formation in electrospinning. (**b**) Merged fluorescent microscopy images of fibers collected at distances of 3 cm, 5 cm, and 10 cm, illustrating core-driven fiber formation. (**c**) SEM images depicting the typical features of coaxial fibers. (**d**) A compilation of sketches, fluorescent merged with optical images, and SEM images to detail the process of sub-Taylor cone formation.

Figure 2 exhibits the distinctive characteristics observed during the coaxial electrospinning process utilizing a fish-source gelatin-based formula. The photograph of a nanofiber captured during coaxial electrospinning (Figure 2a) clearly showed fiber elongation from a single Taylor cone, branching out like a ‘fishbone’ during electrospinning, indicating sub-Taylor cone formation. A longer ohmic flow accompanied by the lower ejection speed of the coaxial fiber was observed. This ohmic flow was further investigated Figure 2b), indicating that the fiber formation was predominantly driven via the gelatin core. This dynamic interplay was revealed through the collection and fluorescent microscopy examination of fibers at various distances. Initially, the ohmic PCL shell encapsulated a highly convective gelatin core, indicating the dominant driving force from the core. As the coaxial fiber elongated, the gelatin core started to transfer its convective energy to the shell, resulting in an unstable ohmic flow of the entire coaxial fiber, presenting as a weak wiping. However, precise fiber deposition could be achieved using an earthed point collector. Dual-scale fibers with nanoscale and microscale dimensions are undesirable (Figure 2c), with 120 *w*/*v*% gelatin as the core and 20 *w*/*v*% PCL as the shell at a 1/2.5 *v*/*v* feed rate. This resulted in 8.54 ± 2.21 μm coaxial microscale fibers and 0.64 ± 0.47 μm nanofibers originating from sub-Taylor cones. The thicker fibers represented core–shell structures, elongated from the Taylor cone, whereas the thinner fibers, indicative of non-core/shell structures, arose from sub-Taylor cones of the shell. The structure of this coaxial fiber was further demonstrated in an SEM image, which focused on a cryo-breakpoint in a coaxial fiber, revealing a solidified ‘cap’ end (Figure 2c). The ‘cap’ formation could be attributed to the high-viscosity gelatin core solution, emerging from the breakpoint, which rapidly solidifies upon contact with air due to swift water evaporation and increased surface gelatin concentration, preventing further evaporation. For drug release, the gelatin solidified cap can easily dissolve in water. After immersion in PBS, the breakdown point revealed a hollow structure, indicating the evacuation of the core solution. The sub-Taylor cone formation process was captured using SEM (Figure 2d). In the initial stage of sub-Taylor cone formation, uneven, thick PCL shells and protrusions were observed under a microscope, with the formation of a conical shape based on the PCL shell surface clearly visible under SEM. In the second stage, the jet initiation was observable under both the microscope and the SEM. Finally, fiber stretching and thinning arose from the PCL shell, which can also be seen in both microscopic and SEM images. This was attributed to the uneven charge distribution [39] (1.3).

**Figure 3:**
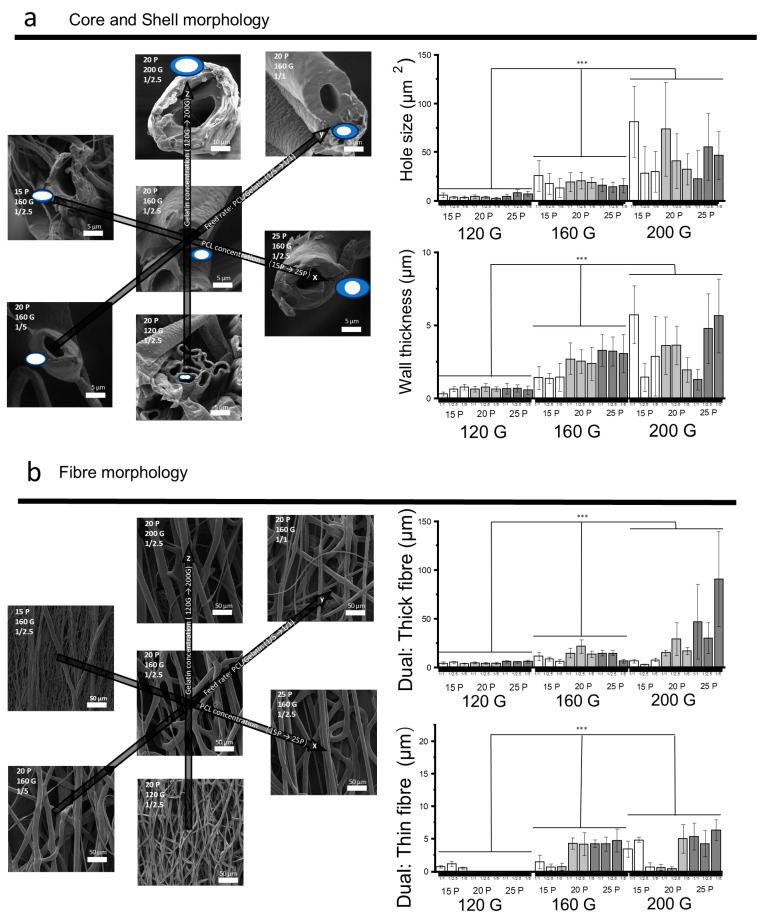
The effects of PCL concentration, gelatin concentration, and the feed ratio on (**a**) fiber pore structure and (**b**) fiber morphology. This figure shows the influence of three parameters, each tested at three different levels, on the hollow structure and fiber morphology. The experimental conditions and outcomes are organized along three axes for clarity. For (**a**) The X-axis represents PCL concentration, incrementally increasing from 15 *w*/*v*% (15P) to 25 *w*/*v*% (25P). The Y-axis details the feed rate ratio of PCL to gelatin, with variations set at 1/5, 1/2.5, and 1/1. The Z-axis tracks gelatin concentration, 120 *w*/*v*% (120G), 160 *w*/*v*% (160G), and 200 *w*/*v*% (200G). For (**b**) the The X-axis represents the group combinations. Statistical significance is denoted by ‘***’, indicating a *p*-value of less than 0.001.

To optimize electrospinning conditions to minimize sub-Taylor cone formation and ensure the stable fabrication of core-shell nanofibers, a three-factor, three-level experiment was conducted. Coaxial fibers were successfully fabricated within the concentration range of 15 *w*/*v*% to 25 *w*/*v*% for PCL/chloroform/DMF as the shell and 120 *w*/*v*% to 200 *w*/*v*% for gelatin/PBS as the core Figure 3 demonstrates the influence of variable gelatin concentrations, PCL concentrations, and feed rates on the hollow structure and recaps fiber morphology patterns. For example, at the chosen parameters of 20 *w*/*v*% PCL, 160 *w*/*v*% gelatin, and a 1/2.5 feed rate of PCL to gelatin, a cross-sectional morphology of PCL/gelatin coaxial fiber was recorded, showcasing an elliptical pore size averaging 20.79 ± 8.78 μm^2^ and surrounded by a uniformly thick PCL shell measuring 2.54 ± 0.82 μm (Figure 3a). Among these parameters, gelatin concentration is the dominant factor influencing the hollow structure and fiber diameter. There was a significant rise in both pore size and wall thickness concomitant with the increase in gelatin concentration. Wall thickness increased from 0.74 ± 0.25 μm to 3.62 ± 1.33 μm, and pore size swelled from 7.89 ± 3.38 μm^2^ to 40.64 ± 26.96 μm^2^ as the gelatin concentration ascended from 120 *w*/*v*% to 160 *w*/*v*%. Interestingly, all combinations featuring a gelatin concentration of 120 *w*/*v*% manifested a bending elliptical pore shape, which could be attributed to the air pressure arising from rapid evaporation [40] (1.3). The PCL concentration influenced the pore shape, with higher concentrations yielding a more circular form, and the wall thickness increased from 1.36 ± 0.36 μm to 3.23 ± 1.00 μm as the PCL concentration was elevated from 15 *w*/*v*% to 25 *w*/*v*%. The feed rate predominantly affected the wall thickness, which expanded from 1.44 ± 0.97 μm to 3.07 ± 1.08 μm. Combinations with 120 *w*/*v*% gelatin and PCL at 20 *w*/*v*% or 25 *w*/*v*% failed to produce thin fibers, and a lower concentration of PCL formed more ratios of the thin fibers, indicating the shell solutions affected the formation of the sub-Taylor cone (Figure 3b). As the gelatin concentration was elevated, so too was the fiber diameter. At a 120 *w*/*v*% gelatin level and a 20 *w*/*v*% PCL concentration with a 1–2.5 feed rate, the average diameter of the thick fibers was 5.46 ± 0.60 μm. Increasing the gelatin to 200 *w*/*v*% saw the diameter of thick fibers surge to 28.99 ± 16.71 μm, alongside the presence of fine fibers measuring 5.01 ± 2.19 μm. Additionally, PCL concentration was found to be directly proportional to fiber thickness. At a lower PCL concentration of 15 *w*/*v*%, the prevalence of finer, nanoscale fibers was noted. For example, at 120 *w*/*v*% gelatin, 15 *w*/*v*% PCL, 1/1 *v*/*v* feed ratio, thin fibers had an average diameter of 0.70 ± 0.19 μm. In contrast, increasing the PCL concentration to 25 *w*/*v*% resulted in a higher average fiber diameter across the board, as observed in the 200 *w*/*v*% gelatin, 25 *w*/*v*% PCL, and 1/5 *v*/*v* ratio group. It was noted that the coaxial fiber fabricated at the 160 *w*/*v*% gelatin, 20 *w*/*v*% PCL, 1/2.5 *v*/*v* feed ratio generated more stable coaxial fiber elongation, with a limited sub-Taylor cone, and was, therefore, selected as the optimized parameters for the following test, including liposomes encapsulation and biocompatibility.

#### 3.1.1. Release Profiles

**Figure 4:**
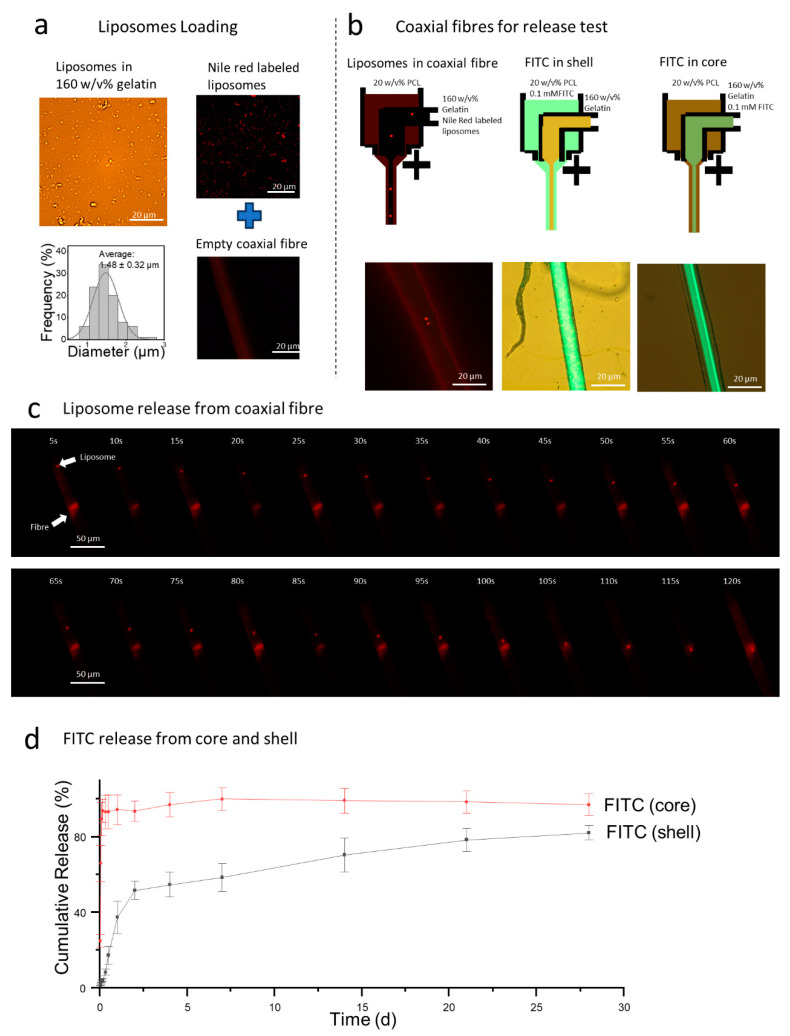
The release profiles of liposomes and FITC from electrospun PCL shell/gelatin core coaxial fibers, prepared with 160 *w*/*v*% gelatin, 20 *w*/*v*% PCL, and a 1/2.5 feed ratio. (**a**) Nile Red-labeled liposome images captured via fluorescent microscope in optical and TRITC modes. (**b**) Sketches and microscope images of the coaxial fiber for release test for liposomes encapsulated in the coaxial fiber, FITC embedded in gelatin core, and PCL shell. (**c**) Time-lapse images illustrating the rapid evacuation of liposomes from the scaffold. (**d**) The FITC release profiles from the core and shell.

Liposomes, with an average diameter of 1.48 ± 0.32 μm, were dispersed within the 160 *w*/*v*% gelatin/PBS solution, indicating their stability within the core solution (Figure 4a).

When labeled with Nile Red, the liposomes exhibited distinct light red fluorescence. Despite the PCL fiber showing some background fluorescence, the successful encapsulation of liposomes within the coaxial fiber was evident (Figure 4b). Rapid release of liposomes was observed when the coaxial fiber was exposed to an aqueous environment (Figure 4c). In PBS, time-lapse images captured the release process of Nile Red-labeled liposomes, which was driven by the dissolution of the solidified gelatin ‘cap’. Initially, liposome movement was slow but accelerated as liquid gelatin was expelled from the PCL shell due to concentration differences, leading to their eventual release outside the fiber. FITC, used as a drug model, was embedded in both the PCL shell and the gelatin core at a concentration of 0.1 mM (Figure 4d). A burst release of FITC was observed within the first hour, with 93.5% of the FITC released from the core into the environment. For the FITC in the shell, half was released after two days in PBS, and by 28 days, a total of 82.2 ± 3.9% of FITC had been released. This difference in release profiles can be attributed to the solubility [41,42], in which gelatin is soluble in water, causing FITC to be released rapidly with the gelatin, whereas FITC embedded in PCL is hindered by the crystalline and amorphous blocks of the PCL, resulting in a slower release.

#### 3.1.2. Liposomes Loaded Coaxial Fiber Characterization and In Vitro Evaluation

**Figure 5:**
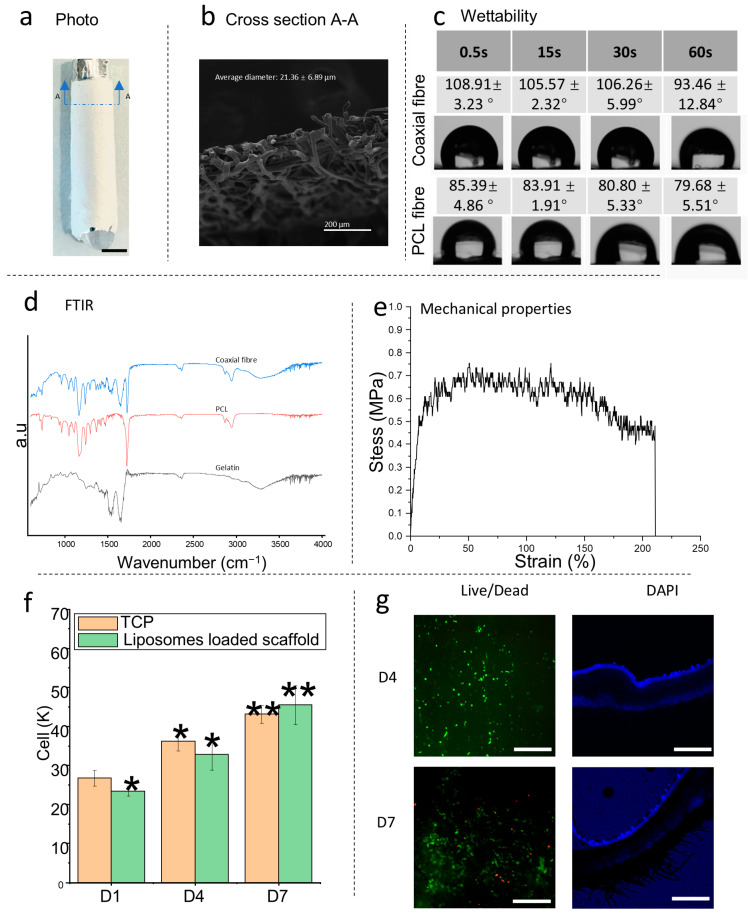
Characterization of the liposome-loaded fibers, showcasing (**a**) a photograph, (**b**) the cross-sectional details (marked as a blue line in (**a**)) via SEM images. (**c**) The wettability of the coaxial fiber and the net PCL fiber, with typical images captured at time points of 0.5, 15, 30, and 60 s. (**d**) FTIR spectra, and (**e**) the typical stress–strain. (**f**) Cell proliferation measured over 7 days, assessed via the PrestoBlue assay. (**g**) Live/dead (left, live green, dead red) and DAPI (right, cell nuclei blue) staining results on days 1, 3, and 7. Statistical annotations are as follows: ‘*, **’ indicates *p* < 0.05 and 0.01 < *p* < 0.05. The scale bar for the fluorescent images represents 200 μm.

The morphology of the coaxial liposome-loaded fiber is illustrated in Figure 5a,b, exhibiting a layer of coaxial fibrous matrix. The hydrophobic property of the PCL shell significantly influenced the wettability (Figure 5c), with the initial water contact angle measured at 85.39 ± 4.86 for neat PCL and 108.91 ± 3.23° for coaxial fiber, demonstrating a minor reduction within the first minute in both groups. The FTIR spectrum (Figure 5d) showed a broad peak from amine groups between 1600 and 1700 cm^−1^ for gelatin [43,44], while the PCL is represented by hydrophobic bonds such as C–C stretching at 1109 cm^−1^ and 1164 cm^−1^, O–C–C stretching at 1193 cm^−1^, and carbonyl stretching at 1723 cm^−1^ and 1737 cm^−1^ [45,46]. As a result, the gelatin/PCL coaxial fiber exhibited peaks for both amine and O–C–C stretching. Due to the low density of liposomes, no separate peak for P=O at 1157 cm^−1^ [47,48] was observed, as it could be attributed to the overlap by the C–C peak at 1164 cm^−1^. The typical stress–strain curve revealed the mechanical behavior of the gelatin/PCL coaxial fiber, with an initial linear elastic region exhibiting a Young’s modulus of 5.2 ± 2.2 MPa (Figure 5e), similar to what Lin et al. reported, a PCL/gelatin coaxial fiber with an organic solvent-based formula exhibited a Young’s modulus of 5 MPa [49]. This was followed by plastic deformation after yielding at 0.7 ± 0.1 MPa, before fracturing at 270 ± 44%, indicating good flexibility.

Cell attachment and proliferation were investigated in this study. It was observed that at D1, 78% of HUVECs attached, slightly lower than that on the TCP (Figure 5f). However, HUVECs proliferated on both the coaxial fiber and TCP, with counts approximating 45 K. Remarkably, cells on the coaxial fiber matrix demonstrated a higher count by D7, likely attributable to the expanded culture space provided by the fibrous matrix. Staining results confirmed the effective seeding and proliferation of cells over the 7 days. Specifically, DAPI staining of the cross-sections highlighted cell adhesion and proliferation with the fibrous matrix made of coaxial fiber.

## 4. Discussion

In this study, fish gelatin/PBS was explored in electrospinning. It kept fluidity even over concentration of 200 *w*/*v*%. Significant reduction in surface tension was observed with fish gelatin/PBS concentrations exceeding 80 *w*/*v*%, which significantly improved its spinnability during electrospinning. Because of this, fish gelatin/PBS was proposed as the core solution in a coaxial electrospinning formula for liposome loading. By leveraging the properties of reduced surface tension at high concentrations and the superior spinnability of gelatin at high concentrations, a coaxial fiber was successfully prepared using a fish gelatin/PBS solution as the core and PCL/chloroform/DMF as the shell solution. This study introduced a new type of gelatin for coaxial electrospinning formulations, successfully loaded structurally intact liposomes, and enabled the topical release of liposomes.

The exceptional fluidity of the fish gelatin/PBS at room temperature proved pivotal in addressing the gelation challenges. This fluidity was inferred in 1961, when Harrington reported that the fish gelatin molecule (dogfish) folded into a trip poly-L-proline II helix in aqueous solution [36,37]. This structure, when compared with calf gelatin, exhibited a lower degree of folding. The application of aqueous fish gelatin solutions in electrospinning has been sporadically reported. Hamid (2010) [50] made the first report, building gelatin shell/PEO core fibers, and later Kwak (2017) linked the low amount of proline in cold water fish gelatin with its fluidity, reporting the fabrication of ultra-fine fibers from 90 to 200 nm using concentrations in the range of 23.7 *w*/*v*% to 35.5 *w*/*v*% [38]. In this study, concentrations exceeding 40 *w*/*v*% were explored, and a reduction in surface tension was observed around 80 *w*/*v*%

The reduction in the surface tension is key for enhancing spinnability and enabling the following development of the coaxial electrospinning formula. For example, the reduction in surface tension (Figure 1c) at around 80 *w*/*v*% enabled fabricating nanofibers at a relatively rapid feed rate. Concentrations up to 200 *w*/*v*% were also tested, producing microfibers at a feed rate of 2.5 mL/h. This reduction trend in surface tension of a gelatin/PBS solution was previously reported in dilute gelatin solutions [51,52,53,54], with regard to molecular rearrangement at the air–liquid interface for a typical colloid solution [51]. This study confirmed this trend at a larger concentration range. Based on the reduction in surface tension and exceptional fluidity, this formulation offers a novel approach to developing a gelatin/PBS-based coaxial formula for liposome loading. Compared to conventional methods that use organic solvents or acids to maintain the fluidity of the gelatin core solution [31,32,33], this approach solves concerns regarding liposome structural instability.

The cohesive force at the interfaces between the air, the PCL shell, and the gelatin core plays an important role in coaxial structure formation. A difference in evaporation at the interfaces led to a buckling structure (Figure 3a, bottom) due to the pressure [40]. The buckling tendency from the shell can be resisted by the cohesive force, such as intermolecular hydrogen bonding within the high-concentration gelatin core solution (Figure 3a, top), which is attributed to the abundant hydroxyl and amine groups in gelatin [23,28]. As electrospinning progresses, the high surface energy and charge accumulation at the Taylor cone cause convective flow within the gelatin core, which subsequently influences the surrounding PCL shell. Due to the interfacial tension between the hydrophilic core and hydrophobic shell, the convective energy transfer leads to stretching and deformation of the PCL shell, aiding in the formation of a stable coaxial fiber structure

The compatibility between gelatin and liposomes is another key point for the successful encapsulation of structurally intact liposomes. This could be attributed to the non-covalent attachment between gelatin and liposomes due to the enriched functional groups, such as amino, carboxyl, and hydroxyl groups in gelatin and phosphates, carbonyls, and polar head groups in liposomes, preventing aggregation via the steric effect [55,56]. These interactions among liposomes and storage solution or matrix were also commonly reported when using natural materials such as hyaluronic acid and chitosan [57].

The structural integrity of liposomes benefits from the non-covalent solidified gelatin cap at the end of the fiber, sealing the liquid gelatin inside. Therefore, compared with previous work [17,18,19,20,21,22], liposome storage within a liquid core solution sealed in the coaxial fiber. Therefore, a burst evacuation of liposomes was observed once liposomes-loaded coaxial fibers were exposed to aqueous solutions, (Figure 4c) due to the dissolution of the cap.

Concentration ranges for PCL from 15 *w*/*v*% to 25 *w*/*v*% and for gelatin from 120 *w*/*v*% to 200 *w*/*v*% can be electrospun to form a coaxial structure. The immiscible solutions (chloroform and water) facilitate a clear boundary between the core and shell. A higher surface tension in the shell benefits the stable formation of the core-shell structure [58,59,60]. Conversely, higher conductivity in the core solution could hinder the formation of the coaxial fiber due to the uneven distribution of charges within the polymer jet. For example, an intact wrapping structure could be achieved via gravity force at a concentration of 10 *w*/*v*% PCL and 80 *w*/*v*% gelatin. However, when an 18 kV voltage was applied, leakage of the core was observed. In this experiment, a high PCL concentration and viscosity were attributed to help offset this instability, thereby stabilizing the coaxial polymer jet.

During the development of the coaxial electrospinning formula, several limitations were identified. The rapid solidification of the fish gelatin/PBS solution at the air–liquid interface interfered with the viscometer sensor vibration, causing it to exceed its upper detection limit. Additionally, while DMF was used to enhance electrospinning performance by improving conductivity, the residual in the PCL shell should be considered for drug-related applications.

Understanding the formation of the sub-Taylor cone is important for avoiding it, as it is a sign of phase separation and not favorable in the electrospinning process. In this study, the formation process of the sub-Taylor cone was captured and analyzed, which was attributed to the accumulation of charges. In detail, the extensive phase separation at the core/shell interface led to polymer-rich and solvent-rich areas in the shell solutions [39], generating a sub-Taylor cone due to the solvent carrying more charges than the solute. Therefore, a sub-Taylor cone was generated in solvent-rich areas and could generate finer fibers at lower concentrations of polymer, which remained consistent with observation. For example, 15 *w*/*v*% PCL/Chloroform/DMF electrospun 1.60 ± 0.65 μm fiber, while its sub-Taylor cone electrospun finer fiber with 0.64 ± 0.47 μm. Interestingly, a distinct relationship between the fiber diameter and the sub-Taylor cone formation was observed. No sub-Taylor cone was observed with the combination of higher concentrations of PCL (20 and 25 *w*/*v*%) with 120 *w*/*v*% gelatin, producing fibers ranging from 4.20 ± 0.99 μm to 6.10 ± 1.20 μm. Higher solution concentration correlated with a larger angle of the Taylor cone [61] and insufficient space prevented sub-Taylor formation on the shell surface.

Overall, the innovative formula developed in this work, incorporating fish gelatin, holds significant promise for advancing liposome delivery technologies. In the future, the electrohydrodynamic writing capability of this formula will be further explored since its ohmic flow can be precisely controlled via the earthed point collector. Also, extended functional materials, such as growth factors and various drugs, will be introduced and encapsulated in the core solution, and could, therefore, develop more sophisticated strategies.

## 5. Conclusions

In this study, a novel coaxial electrospinning formula using fish gelatin aqueous solutions as the core and PCL as the shell was developed, which was used for encapsulating liposomes. This tuneable core-shell structure also allowed for the loading of drugs, enabling dual-release profiles with release times spanning from 1 h to 1 month. Notably, at high concentrations (>120 *w*/*v*%), the gelatin core solution remained liquid not only in the syringe at room temperature but also in the shell structure, which enables the stabilization of liposomes. The core gelatin solution also solidified at the fiber break ends, ensuring the liposome was sealed in the core. These solidified ends easily dissolved in aqueous media, facilitating the rapid local release of liposomes. This innovative formula, alongside its capability for encapsulation and rapid release of liposomes, demonstrates a novel strategy for future advancing scaffold development.

## Figures and Tables

**Table 1:** The investigated electrospinning factors and their level.

Factor 1	Factor 2	Factor 3
PCL Concentration (*w*/*v*%)	Gelatin Concentration (*w*/*v*%)	Feed Ratio (Gelatin/PCL, *v*/*v*)
15	120	1/1
20	160	1/2.5
25	200	1/5

## Data Availability

The original contributions presented in this study are included in the article. Further inquiries can be directed to the corresponding author.
